# Supplementary figures and images for: AI-driven drug discovery using a context-aware hybrid model to optimize drug-target interactions
Source: Sci Rep. 2025 Oct 13;15:35719. doi: 10.1038/s41598-025-19593-4 (PMC12518806; doi:10.1038/s41598-025-19593-4)

## Slide 1
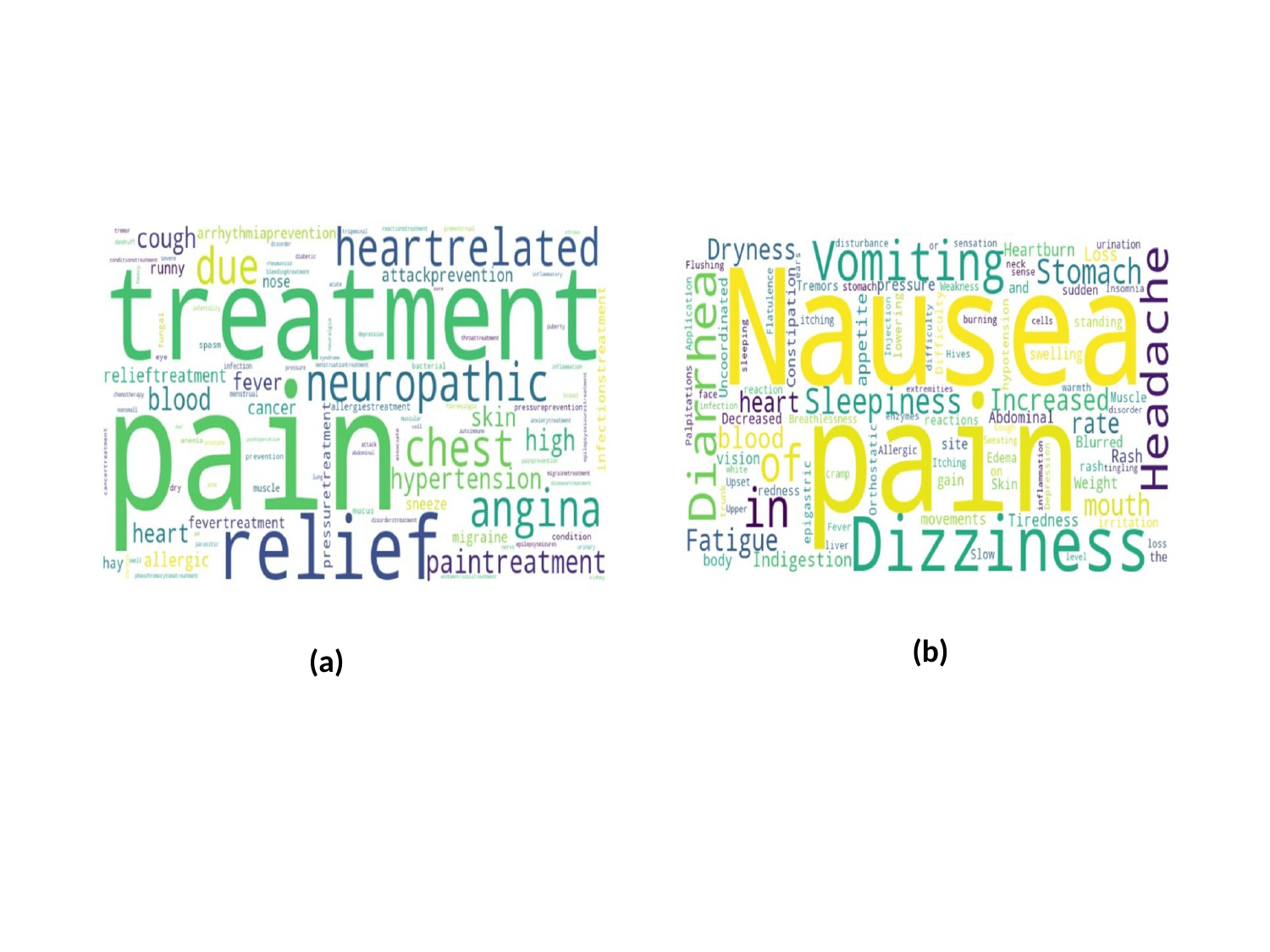

(b)
(a)

## Slide 2
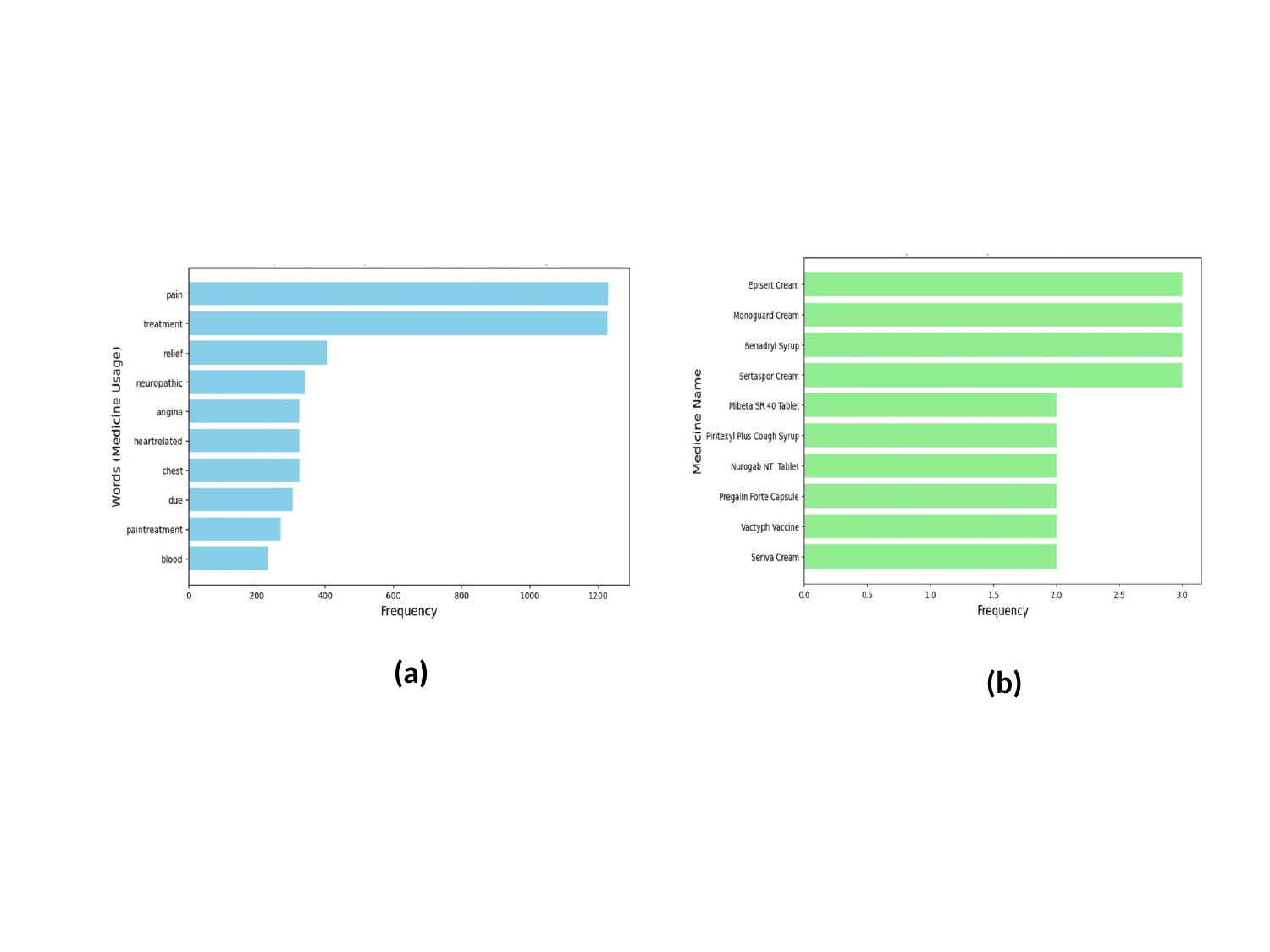

(a)
(b)

## Slide 3
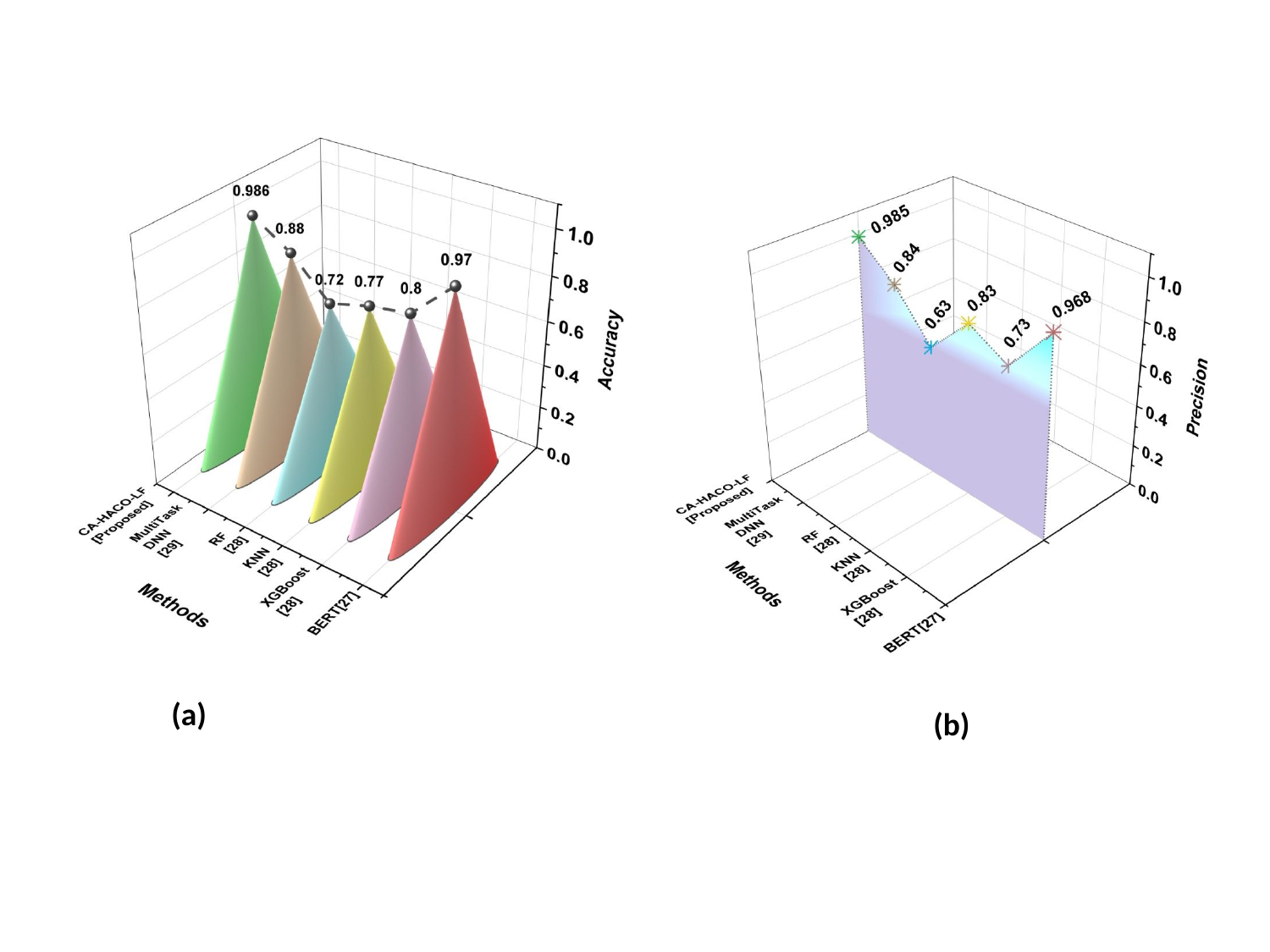

(a)
(b)

## Slide 4
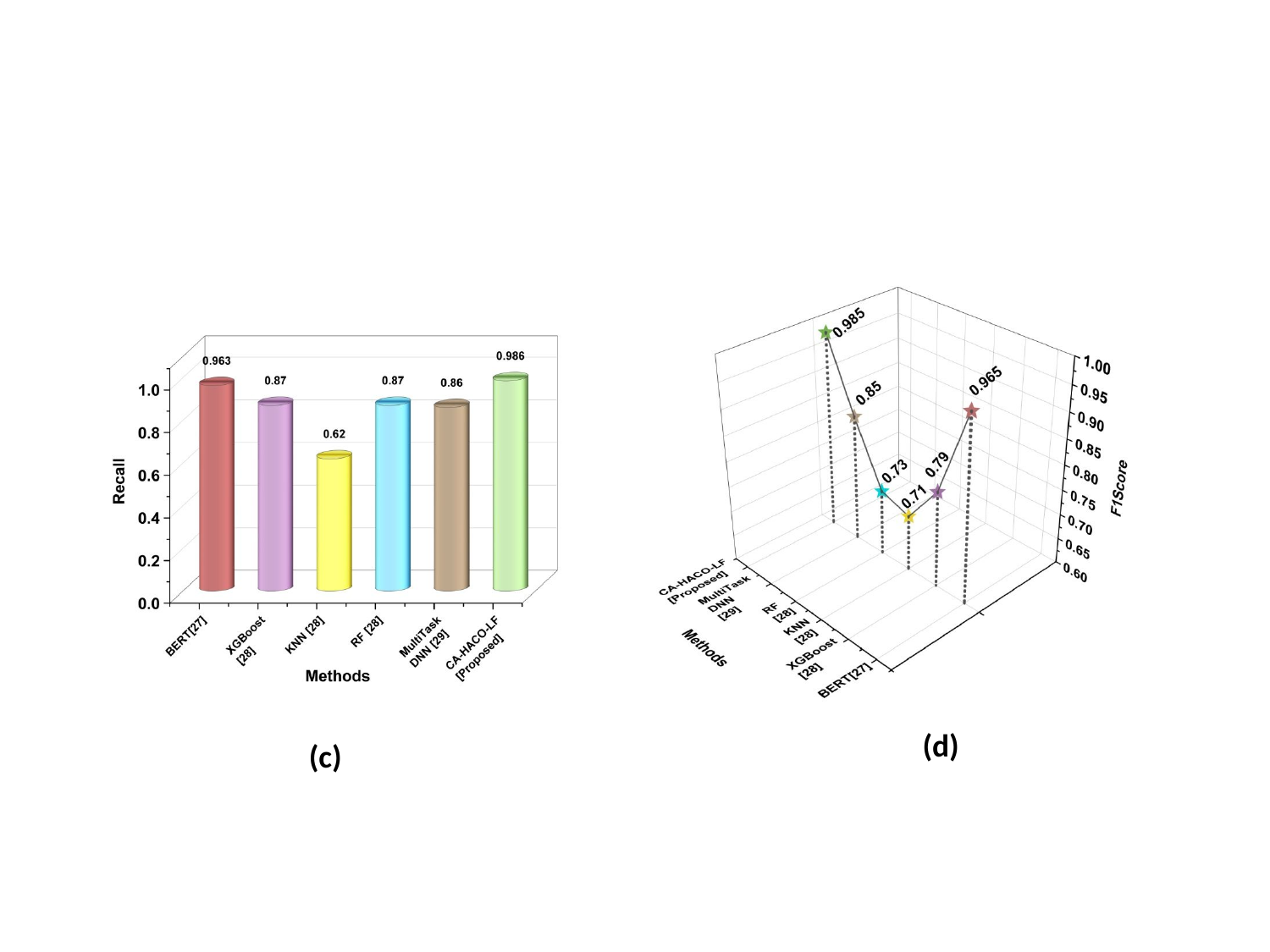

(d)
(c)

Supplement: Supplementary file 2 — Supplementary Material 2 [file 41598_2025_19593_MOESM2_ESM.pptx]
