# Supplementary material for: AI-driven drug discovery using a context-aware hybrid model to optimize drug-target interactions
Source: Sci Rep. 2025 Oct 13;15:35719. doi: 10.1038/s41598-025-19593-4 (PMC12518806; doi:10.1038/s41598-025-19593-4)
Supplement: Supplementary file 4 — Supplementary Material 4 [file 41598_2025_19593_MOESM4_ESM.pdf]

final final

final_20250722_Paper_v2_01.09.25 (3).docx

My Files

My Files

University

Document Details

Submission ID

trn:oid:::27992:110453565

Submission Date

Sep 1, 2025, 5:05 PM GMT+5:30

Download Date

Sep 1, 2025, 5:08 PM GMT+5:30

File Name

final_20250722_Paper_v2_01.09.25 (3).docx

File Size

3.3 MB

35 Pages

9,336 Words

58,263 Characters

6% Overall Similarity

The combined total of all matches, including overlapping sources, for each database.

Filtered from the Report

- Bibliography

Match Groups

43 Not Cited or Quoted 5%
Matches with neither in-text citation nor quotation marks

11 Missing Quotations 1%
Matches that are still very similar to source material

0 Missing Citation 0%
Matches that have quotation marks, but no in-text citation

0 Cited and Quoted 0%
Matches with in-text citation present, but no quotation marks

Top Sources

3% Internet sources
4% Publications
2% Submitted works (Student Papers)

Integrity Flags

0 Integrity Flags for Review

No suspicious text manipulations found.

Our system's algorithms look deeply at a document for any inconsistencies that would set it apart from a normal submission. If we notice something strange, we flag it for you to review.

A Flag is not necessarily an indicator of a problem. However, we'd recommend you focus your attention there for further review.

Match Groups

43 Not Cited or Quoted 5%
Matches with neither in-text citation nor quotation marks

11 Missing Quotations 1%
Matches that are still very similar to source material

0 Missing Citation 0%
Matches that have quotation marks, but no in-text citation

0 Cited and Quoted 0%
Matches with in-text citation present, but no quotation marks

Top Sources

3% Internet sources
4% Publications
2% Submitted works (Student Papers)

Top Sources

The sources with the highest number of matches within the submission. Overlapping sources will not be displayed.

1	Internet	themultiphysicsjournal.com	<1%
2	Internet	www.mdpi.com	<1%
3	Internet	file.techscience.com	<1%
4	Internet	www.nature.com	<1%
5	Publication	Paola Lecca, Michela Lecca. "Graph embedding and geometric deep learning rele...	<1%
6	Internet	repositorio.uchile.cl	<1%
7	Internet	arrow.tudublin.ie	<1%
8	Internet	pmc.ncbi.nlm.nih.gov	<1%
9	Publication	Pushpa Choudhary, Sambit Satpathy, Arvind Dagur, Dhirendra Kumar Shukla. "Re...	<1%
10	Submitted works	The Robert Gordon University on 2024-01-29	<1%

11 Internet

www2.mdpi.com <1%

12 Publication

Justin Shen, Davesh Valagolam. "A Systematic Implementation of Machine Learni... <1%

13 Submitted works

International College of the Cayman Islands on 2025-07-28 <1%

14 Submitted works

University of Nottingham on 2013-04-26 <1%

15 Internet

mdpi-res.com <1%

16 Publication

Mohammad Reza Keyvanpour, Faraneh Haddadi, Soheila Mehrmolaei. "DTIP-TC2... <1%

17 Publication

Raghu Dhumpati, Archana Sasi, Shaik Johny Basha, Suryanarayana Vadhri, P. Sar... <1%

18 Publication

Varun Malik, Ruchi Mittal, Deepali Gupta, Sapna Juneja, Khalid Mohiuddin, Swati ... <1%

19 Internet

arxiv.org <1%

20 Publication

Lee, Won Il, Jae Young Pyun, Yang Sun Lee, and Sang Woong Lee. "Relative velocit... <1%

21 Internet

bmcbioinformatics.biomedcentral.com <1%

22 Internet

research.tue.nl <1%

23 Publication

Roqia Rateb, Ahmed Adnan Hadi, Venkata Mohit Tamanampudi, Laith Abualigah ... <1%

24 Internet

link.springer.com <1%

25 Publication

"Advanced Intelligent Computing Technology and Applications", Springer Science... <1%

26 Publication

Ajay Kumar, Deepak Dembla, Seema Tinker, Surbhi Bhatia Khan. "Handbook of D... <1%

27 Publication

D. Lakshmi, Ravi Shekhar Tiwari, Rajesh Kumar Dhanaraj, Seifedine Kadry. "Explai... <1%

28 Submitted works

Erasmus University of Rotterdam on 2024-05-03 <1%

29 Publication

Mehdi Ghayoumi. "Generative Adversarial Networks in Practice", CRC Press, 2023 <1%

30 Submitted works

University of Oxford on 2025-03-26 <1%

31 Internet

d288qud2qgn4l3.cloudfront.net <1%

32 Internet

www.ijcaonline.org <1%

33 Publication

Cao Yixin, Li Yongzhi, Wei Lingxi, Zhou Yan, Gao Fei, Yu Qi. "Application status of t... <1%

34 Submitted works

Centre for Technical Support on 2025-08-04 <1%

35 Submitted works

Eastern Mediterranean University on 2013-09-05 <1%

36 Publication

Lecture Notes in Computer Science, 2015. <1%

37 Publication

Mohammad Karamouz, Ali Moridi. "Urban Water Engineering and Management", ... <1%

38 Publication

S. R. Reeja, Bore Gowda, Y. S. Rammohan, Ganesan Prabu Sankar, G. Jayalatha. "E... <1%

39 Publication

Xiaolong Li, Feifan Huang, Haotian Sun, Jiayu He, Seyed Mohamad Javidan, Yianni... <1%

40 Internet

jisis.org <1%

41 Publication

Arnur Jumabekov. "Classification of Pneumonia from CXR scans using an Ensembl... <1%

42 Publication

Sanjeetha Pennada, Jack McAlorum, Marcus Perry, Hamish Dow, Gordon Dobie. "... <1%

AI-Driven Drug Discovery: A Context-Aware Hybrid Model for Optimizing Drug-Target Interactions

Ajay Kumar[1,2], Shashi Kant Gupta[1,3], SeongKi Kim[4,*]
[1]Lincoln University College, Malaysia. Email: ajay.phdcse@gmail.com , raj2008enator@gmail.com
[2]IILM University, Greater Noida, India. Email: ajay.phdcse@gmail.com
23 [3]Adjunct Research Faculty, Centre for Research Impact & Outcome, Chitkara University Institute of Engineering and Technology. Chitkara University, Rajpura, 140401, Punjab, India. Email: raj2008enator@gmail.com
20 [4]Associate Professor, Department of Computer Engineering, Chosun University, Gwangju, 61452, Korea. Email: skkim@chosun.ac.kr
Corresponding author's email: skkim@chosun.ac.kr

ABSTRACT

Drug discovery is a challenging and resource-intensive process characterized by high costs, prolonged development timelines, and regulatory hurdles in the pharmaceutical sector. AI-driven recommendation systems have emerged as an effective approach to enhance candidate selection and optimize drug-target interactions. Typical drug discovery methods
34 **are expensive, time-consuming, and frequently have a high failure rate. The inability to quickly identify suitable drug candidates is a significant challenge due to the lack of effective predictive models. To address these issues, the Context-Aware Hybrid Ant Colony Optimized Logistic Forest (CA-HACO-LF) model is proposed. This model combines ant colony optimization for feature selection with logistic forest classification, improving drug-target interaction prediction. By incorporating context-aware learning, the model enhances adaptability and accuracy in drug discovery applications. The research utilized a Kaggle dataset containing over 11,000 drug details. During pre-processing, techniques such as text normalization (lowercasing, punctuation removal, and elimination of numbers and spaces) were applied. Stop word removal and tokenization ensured meaningful feature extraction, while lemmatization refined the word representations to enhance model performance. Feature extraction was further improved using N-grams and Cosine Similarity to assess the semantic proximity of drug descriptions, aiding the model in identifying relevant drug-target interactions and evaluating textual relevance in context. In the classification phase, the CA-HACO-LF model integrates a customized Ant Colony Optimization-based Random**

Forest (RF) with Logistic Regression (LR) to enhance predictive accuracy in identifying drug-target interactions, leveraging the extracted features and cosine similarity for better
42 **performance. The implementation is performed using Python for feature extraction, similarity measurement, and classification. The proposed CA-HACO-LF model outperforms existing methods, demonstrating superior performance across various**
9 **metrics, including accuracy (0.986%), precision, recall, F1 Score, RMSE, AUC-ROC, MSE, MAE, F2 Score, and Cohen's Kappa.**

Keywords Drug Discovery, AI-driven Recommendation Systems, Drug-Target Interactions, Ant Colony Optimization, Feature Selection, Logistic Forest Classification, Context-Aware Learning, Cosine Similarity, N-Grams, Model Performance

Introduction

The identification of new potential therapies has been a vital component in enhancing human wellness. Populations of people globally experience numerous difficulties and remain impacted by microorganisms. Also, serious medical conditions like diabetes and cancer have been a persistent threat to human health [1]. Traditional drug development and discovery involves preclinical and clinical trials, lead drug discovery and effectiveness, identifying targets and validation, and other time-consuming, harmful procedures [2]. Reports of significant advances in drug discovery that have been allocated to computational intelligence were frequently reported in
24 the technological and available sectors in recent years [3]. The process of finding new drugs is time-consuming, complex, and dangerous. Partnerships appear to strengthen the possibility of clinical success, expand the target inventory by identifying emerging targets, use undruggable targets, and improve the development of new medications [4].In a desire to gain a greater knowledge of senescent cell biology, *dasatinib (D)* and *quercetin (Q)*, the first senolytic drugs, were discovered in 2015 [5]. Certain genes have been discovered to be essential for the ability to survive cancer cells across the drug development process, and the proteins that encode them were found to be cancer-selective targets [6]. By analyzing a large number of candidate compounds, High-Throughput Screening (HTS) improves the drug identification process. By detecting possible active molecules and eliminating undesirable structures, Virtual Screening (VS) enhances the performance [7]. During the identification of a potential substance, extensive testing in clinical and preclinical investigations is conducted to evaluate its efficiency, safety,

and possible adverse effects [8]. Target verification, compound discovery, improvement, preclinical evaluation, and clinical trials are all involved in the multi-stage process of introducing drugs into medical practice. Historically, this process has required an enormous amount of time and resources, whereas these initiatives continue to result in substantial losses, negative drug effects, and persistent challenges in treating diseases like diabetes and cancer [9]. Most clinical pharmaceutical candidates fail to find the clinical alternative with the required biological impact due to safety or efficacy issues [10].

Problem Statement

The lengthy development cycles, high cost, complexity, and low success rates in establishing effective drug-target interactions provide significant difficulties to the drug discovery process. Large biomedical datasets are difficult to examine effectively using traditional computational approaches and frequently lack the contextual awareness and prediction accuracy required. Finding significant connections between medications and biological targets is further complicated by the existing algorithms' lack of intelligent feature selection and semantic comprehension. To identify the drug-target interactions more quickly and affordably, a strong, context-aware hybrid model is essential. The research develops the CA-HACO-LF method to enhance the drug discovery performance, and it improves the feature extraction, optimizes classification, and increases prediction accuracy.

Objective and Contributions of this Research

Developing a strong, Context-Aware Hybrid Ant Colony Optimized Logistic Forest (CA-HACO-LF) model to improve drug-target interaction prediction in drug discovery is the primary objective. The approach intends to increase the effectiveness and precision of potential drug identification by combining HACO for efficient feature selection with an LF classifier for precise prediction. It aims to integrate CA and increase flexibility across various medical data conditions by utilizing conceptual feature extraction approaches, such as N-Grams and Cosine Similarity.

16

- ✓ The research intends to provide an effective prediction of drug-target interactions in drug discovery.

- ✓ The 11000 Medicine Details dataset was obtained, and the obtained data was preprocessed by certain techniques called text normalization, stop words removal, tokenization, and lemmatization.
- ✓ Processed data are extracted with significant features by utilizing the feature extraction techniques called N-Grams and Cosine Similarity.
- ✓ The CA-HACO-LF method is proposed to enhance the forecasting precision in
38 classifying drug-target interactions.
- ✓ The performance of the proposed and existing techniques is determined by comparing the results through various performance metrics, whereas the performance of the CA-HACO-LF method shows more significance in drug prediction and classification.

The proposed CA-HACO-LF model improves precision medicine, clinical trial selection screening, and medication repurposing, among various applications in pharmaceutical research and development.

Developing, merging, and linking were the three ideas that have been brought to combine the elements into the compound discovered in the research [11]. Variational Autoencoder (VAE) and reinforcement learning models were examined. AI-enabled fragment-based drug discovery techniques make advancements that contribute to the highly efficient exploration of the enormous chemical universe. The Black Box issue with DL models' operation interpretation was the limitation presented in the research.

There was a lot of interest in creating inventive methods to use Deep Learning's (DL) capabilities
22 in low-data conditions, as demonstrated in [12]. From de novo design to protein structure prediction and synthesis planning, DL has become increasingly significant throughout the drug discovery process. The results assumed the risk of predicting potential paths that exist in drug discovery using low-data training. Insufficient criteria and datasets have been developed in the research to standardize the assessment, selection, and creation of creative approaches with a higher potential for drug discovery.

Due to Plasmodium parasites' increasing medication resistance and inability to stop transmission within human hosts, malaria continued as a serious threat to public health, as discussed in [13]. It explored various ML and DL techniques, and the results showed that the Fingerprints and Graph

Neural Networks (FP-GNN) model effectively represented the main structural characteristics in drug discovery. A limitation of the investigation was that it was computationally complicated to perform with the large amount of data.

Three important Transcription Factors (TFs) control the tumor cell-specific regulatory networks that were discovered [14], and the investigation of the protein structure of Clear Cell Renal Carcinoma (ccRCC) allowed research to identify the TF EPAS1/HIF-2α using pharmacological virtual screening. Both the ML technique and a deep Graph Neural Network (GNN) were employed. Certain substances that influenced the therapeutic medication of individuals with ccRCC were identified based on the characteristics of the tumor microenvironment. Drug resistance and variation in tumors significantly limited the effectiveness of cancer treatment.

Experimental screening based on targets and phenotypes has become a popular approach for finding anticancer drugs, whereas these methods were labor-intensive, time-consuming, and expensive [15]. 832 models were constructed using the Fingerprint GNN(FP-GNN) technique to
33 forecast the inhibitory impact of drugs against targeted and tumor cell types. The research did not include tumor cells, and DeepCancer updates to address new targets.

18 The DL-based computer model was used to estimate Drug–Target Affinities (DTAs) by learning drug networks, protein sequences, and dual drug sequences [16]. To specifically enhance the spatial characteristics of drug and protein sequences, layered multilayer squeeze-and-excitation networks were employed. Additionally, compact graph isomorphism networks were employed to collect drug visualizations and differentiate between chemical structures. Experimental assessments using repeated cross-validation on different datasets showed that DoubleSG-DTA consistently performed better than other research. The investigation involved computer predictions without experimental confirmation, which could fail to accurately represent the complex structures and interactions of biological processes.

5 A description of geometric DL's modern applications in bioorganic and medicinal chemistry in
5 the research, with particular attention on the technology's potential for structure-based drug discovery and development, has been provided [17]. The main emphasis was on ligand binding
5 site, balance, and structure-based de novo molecule creation with molecular attribute prediction. The application of physics-inspired techniques and equivariant neural networks to structure-based drug development was not well-established.

Developing a more effective, methodical medication design for periodontitis before clinical trials was the objective [18]. To cure dental disease, the researchers employed DL and systems biology techniques to develop and find new drugs. There was no investigation into possibly beneficial substances in potential drug discovery technologies.

Based on the properties of Traditional Chinese Medicine (TCM) ingredient small molecules and FDA-approved combination medicine data obtained from the DrugCombDB database, a drug combination training set was created utilizing 16 distinctive variables [19]. For the categorization and prediction of combination medications, the RF model was chosen. Clinical trials were lacking to validate the research's findings and the use of TCM in other domains.

A more logical approach to drug discovery by computer-aided drug design and disease-related target discovery, as research advances, has been demonstrated [20]. The identification and forecasting of compound-protein associations were then investigated after the DL model was built using a Recurrent Neural Network (RNN). Its dependence on computer models allowed it to ignore complex pharmacodynamic and pharmacokinetic interactions, and it lacks biological or clinical validation.

30 The ML approach that accurately anticipated the findings of the research using biological activities, the chemicals' physicochemical properties, target-related factors, and compound representation based on Natural Language Processing (NLP) techniques was determined [21]. It provided findings and conclusions from an RF classifier that has an average accuracy of 93%. The lack of private or clinical datasets limited the model's accuracy and generalizability.

11 Using ML techniques, multifunctional antimicrobial compounds and lead compounds that inhibit the antibiotic targets Deoxyribonucleic Acid (DNA) gyrase and Dihydrofolate reductase in
12 Escherichia coli, with 18387 non-specific drug-like chemicals, were introduced in the research
12 [22]. With an accuracy of 0.91, the findings showed that the Gradient Boosting Classifier (GBC) was the most effective in determining a compound's effectiveness against DNA gyrase. There was no demonstration of any other categorization methods for screening antibacterial compounds.

21 A well-known network-based ML technique for the prediction of drug-target and drug-drug interactions and evaluations for predicting network relationships was applied [23]. Following

experimental assessment, the three greatest performers, including RONE, ACT, and LREW5, were identified. The algorithmic prediction capability was impacted by the smaller number of instances.

The prediction model for potential Drug Target Interactions (DTIs) was presented in the research [24]. The unique properties of proteins and medications' structural forms were utilized. Light-Boost and ExtraTree's experimental results utilized the structures and feature data, yielding 98% accuracy. The model's generalizability across a variety of diseases was unclear and untested, and it lacked experimental validation and overfitted due to its great reliability.

Drugs' unpleasant tastes have been evaluated in the final phases of research. The BitterIntense, an ML tool that utilized a molecule's chemical structure to determine whether it's very bitter or not very bitter, was introduced [25]. Using multiple test sets, the model was trained on a variety of chemical substances and exhibited accuracy levels above 80%. To find and create new bioactive chemicals, the research does not incorporate computational taste prediction with other computational technologies.

Structure of the research: Section 1 presents the introduction. Evaluation of drug-relevant
13 articles is provided in Section 2. Research methodology is explored in Section 3. In Section 4, results and discussions are presented. Section 5 determines the conclusions of the research.

Research Methodology

Improving prediction accuracy in detecting drug-target interactions is the objective of the investigation. The Kaggle data with 11,000 drug data points was used in the research. Techniques, including text normalization, were used during pre-processing. Lemmatization improved word representations to improve model performance, while tokenization and stop word removal maintained relevant discovery of features. Utilizing N-grams and Cosine Similarity to evaluate the semantic similarity of drug descriptions, feature extraction was further enhanced. This enabled the model to find pertinent drug-target interactions, along with assessing textual significance in context. Using obtained characteristics and cosine similarity, the CA-HACO-LF model in the classification was used to improve predictive accuracy in finding drug-target interactions.Fig.1. presents a workflow for drug–target identification. It starts with data collection from biological and chemical sources, followed by data preprocessing to clean and

standardize information. Next, feature extraction transforms raw data into meaningful descriptors for drugs and targets. These features are processed through classification models, such as machine learning, to predict possible interactions. Finally, experimental results validate the predictions, ensuring accuracy and reliability. This pipeline supports efficient drug discovery by integrating computational analysis with biological validation.

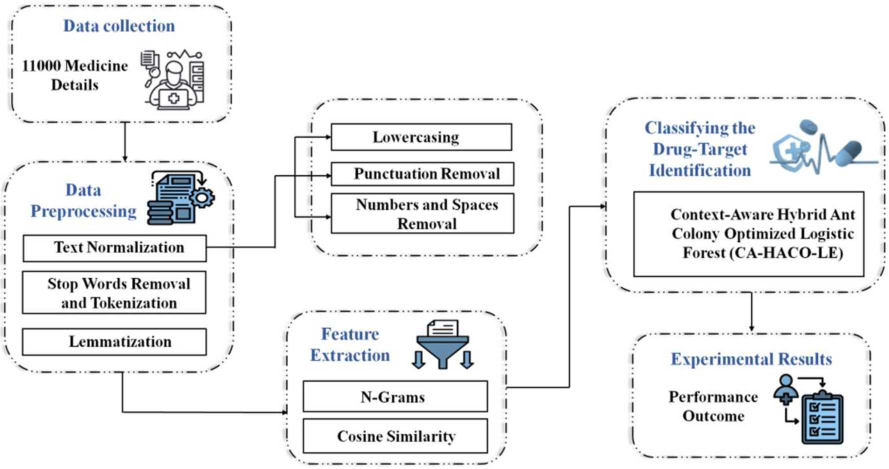

Fig.1 Process Involved in the Research

Data Collection

The 11000 Medicine details dataset [26] is obtained from Kaggle. Medical professionals, data professionals, and supporters desire to discover more about the availability of medications and medical supplies, as they anticipate this dataset to be a useful resource. It has a wealth of data about more than 11,000 medications that were collected from 1mg, a well-known online pharmaceutical and healthcare organization. There are six features, such as medicine name, salt composition, uses, manufacturer, image URLs, and percentage of reviews (Table 1).

Table 1. Feature Descriptions

Features	Descriptions
Medicine name	The dataset is an extensive compilation of pharmaceutical items, containing the names of more than 11,000 medications.
Salt composition	Medical practitioners and academics researching drug interactions and effects depend heavily on the salt content of each medication.
Uses	Discover the illnesses and diseases for which prescription drugs are utilized,

	which are relevant to medical professionals and researchers investigating the effectiveness of treatments.
Manufacturer	The dataset provides useful data about pharmaceutical producers, supporting supply chain management, quality control analysis, and the identification of production patterns.
Image URLs	Drug identification and visual evaluation are made easier by patients having access to visual URLs for each medication.
Review Percentages	The three types of review percentages in this dataset are Excellent, Average, and Poor. These sections provide an extensive description of each medication's ratings and comments.

Data Preprocessing

Preprocessing is the process of converting unprocessed data into the proper structure that can be used for evaluation and ML models. It comprises organizing, modifying, and cleaning data to enhance its quality and utility for further processing. This research provides various data preprocessing techniques, like text normalization, stop word removal, and tokenization, along with lemmatization for processing the data from the dataset.

- ***Text normalization:*** To ensure consistency and suitability for various NLP tasks, text data need to be cleaned and preprocessed through a process known as text normalization. The procedure involves multiple types of processes, including case normalization and punctuation removal, along with number and space removal.

Lowercasing: To maintain the data as consistent, all of the content in the "Uses" category is converted to lowercase, which ensures that variants such as headache are considered interchangeable phrases. **Punctuation Removal:**It excludes symbols and special characters that interfere with text evaluation and keeps texts without unnecessary symbols like!, ?, etc. **Numbers and Spaces Removal:** It eliminates any numerical values that aren't necessarily useful for textual evaluation. It assists in directing attention to medical situations or applications over numbers. It makes data formatting cleaner by eliminating unnecessary preceding or surrounding spaces and keeps the ML model with no whitespace inconsistencies.

- ***Stop word removal and Tokenization:*** Eliminating terms that occur frequently in all of the corpus's records is known as stop word removal. The technique of breaking the text into a collection of significant components is called tokenization, and the components are known as tokens.

Stop word removal: To improve processing efficiency, common words that lack significant meaning to the text, like "the," "or," and "is," are eliminated. **Tokenization:** Separating the information into separate words makes modification and evaluation easier, like "Medicine Name" as "Medicine" and "Name".

- ***Lemmatization:*** Using a lexicon that considers context, it reduces words into the simplest form. Every time, a valid term is returned by the lemmatization.

Before processing the input, the function validates the words in a proper string. When the input value is not a string, it remains unchanged to avoid errors. To keep the cleaned words clear, the processed words are reassembled into a string. Using the processed text, the dataset's "Uses" category is updated.

Feature Extraction

The process of converting unstructured data into a collection of beneficial attributes is called feature extraction, and it involves determining and selecting the most pertinent features from the data and then presenting information in a new and easier-to-manage format. This process includes two significant extraction techniques, such as N-Grams and Cosine Similarity, that extract the significant features from the preprocessed data.

- **N-Grams:** To capture contextual patterns in a given text, N-grams are used to represent sequences of n objects, frequently words or characters. It converts text into numbers, which are utilized in ML algorithms.

Numerical representations of the medications' applications are created from the textual descriptions. An n-gram model is used for this process by capturing word sequences (bigrams and unigrams) that describe the usage pattern of each medication.

- **Cosine Similarity:** In applications like text evaluation, cosine similarity is frequently used to assess the similarity of two vectors. Vectors are considered to be identical if the

cosine similarity is 1, orthogonal (unrelated) if it is 0, and diametrically opposing if it is -1.

To compare the similarities of various medications, the cosine similarity metric is utilized once the text has been transformed into numerical form. The medications are more likely to have equivalent applications while the similarity score is high.

Classifying the Drug-Target Interaction by Employing the Context-Aware Hybrid Ant Colony Optimized Logistic Forest (CA-HACO-LF) Model

Using the obtained characteristics and cosine similarity, the CA-HACO-LF model enhances the predictive accuracy in finding drug-target interactions during the classification process by integrating a specific Random Forest based on Ant Colony Optimization with Logistic Regression.

Context Aware (CA)

The CA is incorporated into the model, which means that it considers the context when the data is generated, along with basic data. When it relates to medication discovery, this necessitates the patient's medical history and symptoms, along with practical elements like the time of day or the patient's location that affect a medicine's effectiveness. Context awareness enables the algorithm to produce more precise and individualized drug recommendation predictions.

Logistic Forest (LF)

The RF and Logistic Regression (LR) are clear and interpretable classifiers, combined as a hybrid LF technique. Through the use of both model capabilities, a hybrid method is introduced to improve classification performance by averaging the model predictions.

LR: One classification approach that makes use of the ensemble methodology is LR. It predicts the probability of categorical outcomes in the drug discovery classification process. Its implementation is feasible, and it will be highly effective, as it produces a model with adequate performance. By integrating an error function into the drug discovery prediction function, it essentially employs an alternative method to the regularization technique used in the proposed model, which estimates the parameters and selects the variables. To produce the model, LR divides the data into sub-spaces that support the main assumption $q < N$, which is required

for logistic regression model training. For $j = 1, 2, \ldots, N$, let a_j be the $q \times 1$ vector of predictions and B_j be a binary outcome variable with two potential values $\{0,1\}$. The LR probability model is provided in Equation (1), with coefficients β_0 and $\beta = (\beta_1, \beta_2, \ldots, \beta_q)^S$. The equation models the probability $Q(B_j|a_j)$ of event B_j given features a_j^S, where β0\beta_0β0 is the intercept, β is the coefficient vector, and f defines the logistic function.

$$Q(B_j|a_j) = \frac{1}{1+f^{-(\beta_0 + a_j^S \beta)}} \quad (1)$$

Since the maximum probability method for estimating β_0 and β has no closed forms, an iterative or numerical approach is employed. The following Equation (2)is used to estimate the parameters using this method.

$$\{\hat{\beta}_0, \hat{\boldsymbol{\beta}}\}^{(l)} = \{\hat{\beta}_0, \hat{\boldsymbol{\beta}}\}^{(l-1)} - \boldsymbol{G}^{-1}(\beta_0^l, \beta^l)\nabla_{\mathrm{k}}(\beta_0^l, \beta^l) \quad (2)$$

In this case, l represents the estimation process's iteration index. The gradient vector, or first derivative of the log-probability function, is represented by the vector $\nabla_{\mathrm{k}}(\beta_0^l, \beta^l)$, and the Hessian matrix for $k(\beta)$ is represented by$\boldsymbol{G}^{-1}(\beta_0^l, \beta^l)$. Combining weak classifiers to create a stronger model is the fundamental idea behind this approach, where each classifier is constructed from the framework using a distinct set of variables. For $n = 1, 2, \ldots, N$, let θ be the space of predictions in the entire data, and θm represents the m-th subspace. The data is initially divided into N sub-spaces using the following algorithmΘ: $\Theta = (\theta_1, \theta_2, \ldots, \theta_N)$. Each subspace has been selected with approximately the same number of members or in balance. The average value will be utilized to collect the prediction values or occurrence probabilities of each model. This threshold is often set at 0.5.LR creates several ensembles to enhance the classifier's performance, and the outcome is determined by a majority decision.

Fig.2. illustrates a single-layer perceptron used in neural networks. Products are combined in the summation block, producing a net input. The net input is then passed through an activation function to add non-linearity, followed by a threshold function to determine the final output. The difference between the predicted output and actual output generates an error, which is used to adjust weights for learning.

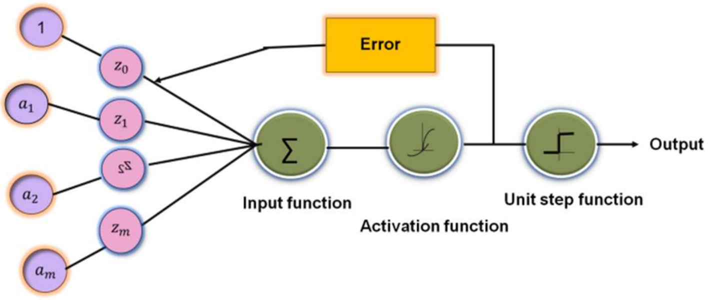

Fig.2. Architecture of LR

RF: A group of classification trees constitutes the RF algorithm, and each tree decides only for the class that is most frequently assigned to the input data. It combines multiple decision trees for better generalization, which enhances the performance in drug discovery forecasts.

10 Fig.3. illustrates an ensemble learning approach for drug dataset analysis. The dataset is divided into subsets and processed through multiple models or classifiers, each producing independent results (Result-1, Result-2, … Result-n). Instead of relying on a single model, the outcomes are combined using a majority voting mechanism, where the most common prediction is selected as the final decision. This technique reduces bias, improves accuracy, and enhances the robustness of drug–target identification by leveraging the collective strength of multiple classifiers.

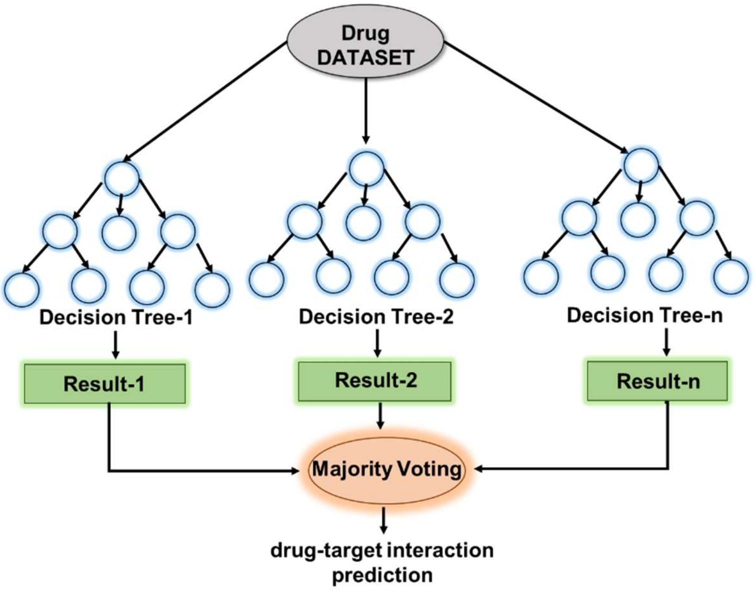

Fig.3. RF Classifier's Basic Architecture

The RF variable's significance factor in the research is determined by observing the sensitivity of
1 sample size, variable amount, responsiveness to various technique parameters, and sensitivity to

1 the existence of correlated variables. As a supervised learning approach, RF creates the Decision
27 Tree's (DT) forest. Its accuracy approaches many ML methods, especially when dealing with large datasets that contain a large number of features. The collecting approach is typically used
1 for training, where each tree is constructed by a randomized selection process from the training data. There was an integration between the variety of classification trees and the RF classifier. Equation (3) is used in the calculation of the classification outcome.

$$D(s) = \max_{Q} F_s \sum_{j=1}^{L}\left(d_j(S) = Q\right) \quad (3)$$

The variable S is the original dataset's training set. The selections from the S dataset are denoted by T and L. Using a random vector, the application automatically creates L decision trees for every subset. The classification result is represented by $D(s)$, while the classification result of the j^{Th} decision tree is shown by$d_j(S)$. The target category in the present instance is Q. However,
1 a number of RF hyperparameters are used to speed up the procedure or improve the model's drug discovery prediction abilities. When managing high-dimensional data, RF achieves superior performance by performing an implicit feature selection procedure. In determining feature importance in RF, the Gini importance is frequently utilized as a measurement factor. As a result, the relevance ratings contribute to determining the importance of DTs to the classifier. Equation (4)The functionmeasures performance, where increasing error values (e_1,e_o) decrease the overall score, and minimizing these errors maximizes $j(s)$.

$$j(s) = 1 - e_1^2 - e_0^2 \quad (4)$$

Variable e is the proportion of the classes. The class is represented by $i = 0{,}1$, and e_i in Equation (5) is the proportion of N_i samples that represent the total number of samples N.

$$e_i = \frac{N_i}{N} \quad (5)$$

A threshold on variable Θ is used to split and send products to two distinct sub-notes (s_q& s_p), which results in diminishing δi.The process is expressed in equation (6).Here, e_q and e_p are coefficients (weights) that scale the values of j at those reference points.

Thus, $\delta j(s)$ measures the residual or correction term after accounting for the influence of s_q and s_p on$j(s)$.

$$\delta j(s) = j(s) - e_q j(s_q) - e_p j(s_p) \tag{6}$$

Then, using all-inclusive values of Θ that are available in the node's overall thresholds, an exhaustive search is conducted. Equation (7) is used to preserve the decreases in Gini impurity values for each variable independently by taking all nodes s.

$$J_H(\Theta) = \sum_o \sum_p \delta j_\Theta\,(s, S) \tag{7}$$

Variable J_H considers the frequency that featuresΘ is chosen at a split and the significance to the classifier for a specific assignment.It aggregates the contribution of different outcomes (o) and parameters (p) by summing their associated error or reward term δj_Θ. The weight of each classifier is dynamically changed according to the importance of the symptom, as an alternative to relying on the standard majority voting approach. For instance, the RF model, weighted more heavily in the instance of cancer data, is more complex. Both classifiers independently produce predictions. By combining the forecasts using a majority vote mechanism, the more frequent prediction among the two models determines the outcome.

32

Hybrid Ant Colony Optimization (HACO)

The Ant Colony Optimization (ACO) algorithm is a metaheuristic that derives inspiration from the actual ants finding the quickest route to sources of data. Ants frequently utilize the routes with the highest concentration of pheromones. An important agent-based system that mimics ants' natural behavior, including the cooperative and adaptive mechanisms, is the ACO algorithm. The ACO algorithm quickly determines the shortest route from the data source to the habitat and vice versa by simulating the methods employed by real ants without the need for visual cues. The ACO algorithm generates solutions across a period of cycles, or iterations. Several ants use heuristic knowledge and the collective knowledge of earlier ant groups to provide comprehensive solutions in iterations. The main factor contributing to the ACO algorithm's shortcomings is the occurrence of inactivity. To address this issue in the ACO algorithm, the HACO is suggested in the research. HACO facilitates the selection of the most relevant features for a classification process in drug delivery. Various stages presented in the optimization approach are explained below.

Dynamic Motions of Ants: It is suggested that the dynamical movements probability rule, which combines deterministic and random selection, improves the global search strategy's

selection process. Throughout the evolutionary process, the phenomenon of the route is changing. Since symptoms change over time, the research dynamically adjusts the pheromone strength based on the significance of features compared to employing fixed pheromone levels. For instance, with time, certain traits (such as medical records or the intensity of symptoms) get increasingly important for classifying diseases. Pheromone updating rules are based on domain-specific knowledge, such as symptom correlations. For example, flu and pneumonia co-occur with symptoms including fever and cough. This is able to select more pertinent elements. The possibility of travelling to the following destinations, denoted as Q_{ji}^{l}, is shown in Equations (8-9).

The objective function $JH(\Theta)$ used in the CA-HACO-LF model, where ant colony optimization (ACO) guides the selection probability of feature paths. The transition probability $Q_{ji}^{l}(s)$ depends on pheromone intensity (τ), heuristic information (η), and adaptive factor a_{ji}, normalized over all allowed paths. It balances dataset size and path distance, ensuring optimal feature relevance and improved classification accuracy.

$$Q_{ji}^{l}(s) = \begin{cases} \frac{\tau_{ji}(s)^{\alpha} \times \eta_{ji}(s)^{\beta} \times a_{ji}(s)}{\sum_{l \in allowed_l} \tau_{jl}(s)^{\alpha} \times \eta_{jl}(s)^{\beta} \times a(s)} & if\ i \in allowed_l \\ 0 & Otherwise \end{cases} \quad (8)$$

$$a_{ji} = \frac{M \times S_d}{M \times S_d + \delta \times P_d(j,i) \times \eta(j,i) / \eta_{Maxi}} \quad (9)$$

Where M represents the number of ants, S_d is the number of iterations that are presently occurring, and max is the heuristic function's maximum value(j, i). From the start of the first iteration, $P_d(j, i)$represents the total ant quantities that traversed with route(j, i). ParametersP_d and $\eta$are similarly taken into consideration by the a_{ji}. While the phenomenon's effect on the inadequate solution continued to improve, the quantity of ants and the component $a_{ji}$are reduced as the iteration went toward the inadequate solution.

Updated Rules: The residual phenomena are updated following each search to avoid encapsulating the predictive factor by the residual pheromone information. Description of the phenomena updates approach using the HACO algorithm's expensive approach is determined in Equations (10-13).

39

Equation 10 represents the pheromone update rule, where $\tau_{ji}(s+1)$ is the pheromone level on edge ji at the next iteration. ρ is the pheromone evaporation rate, $\tau_{ji}(s)+\Delta\tau_{ji}$ is the current pheromone level, $\Delta\tau_{ji}$ is the pheromone deposited by all ants during the iteration, and $+\Delta{\tau_{ji}}^{*}$ is the pheromone reinforcement from the best solution.

$$\tau_{ji}(s+1) = \rho\tau_{ji}(s) + \Delta\tau_{ji} + \Delta{\tau_{ji}}^{*} \tag{10}$$

Equation (11), $\Delta{\tau_{ji}}^{*}$is the total pheromone deposited on edge (j,i) by all MMM ants, where $\Delta{\tau_{ji}}^{l}$denotes the pheromone contribution from the l^{th}ant.

$$\Delta{\tau_{ji}}^{*} = \sum_{l=1}^{M} \Delta{\tau_{ji}}^{l} \tag{11}$$

Equation (12) represents the pheromone deposited by the l^{th}ant is P/K_L, where P is a constant pheromone strength and K_L is the length or cost of the path chosen by ant l; if the edge is not visited, no pheromone is added.

$$\Delta\tau^{l} = \begin{cases} P/K_L\,, Pass\ path(j,i) by\ ant\ l\ in\ the\ iteration \\ \qquad 0, \qquad\qquad\qquad other \end{cases} \tag{12}$$

Equation (13), $\Delta\tau^{*}$ is the additional pheromone reinforcement, where δ is a scaling factor, P is pheromone strength, and L^{*} is the length or cost of the best solution; otherwise, no reinforcement is applied.

$$\Delta\tau^{*} = \begin{cases} \delta\, P/L^{*}\,, the\ Edge(j,i) j\ is\ one\ of\ the\ found\ optimal\ solutions \\ \qquad 0, \qquad\qquad\qquad other \end{cases} \tag{13}$$

Adaptive Adjustment Approach: To create a generally uniform phenomena distribution and successfully resolve the conflict between widening the search and finding the best solution, an adaptive phenomena adjustment technique is presented to identify the local optimal solution. In the adjusting phenomena $\Delta{\tau_{ji}}^{l} = P(s)/K_L$, the constant of phenomena intensity P is chosen to be replaced by the real variable function $P(s)$ (Equations (14-15)).

$$\Delta{\tau_{ji}}^{l}(s) = P(s)/K_L \tag{14}$$

$$P(s) = \begin{cases} P_1 \qquad\quad s \le S_1 \\ \quad P_2 S_1 < s \le S_2 \\ \quad P_3 S_2 < s \le S_3 \end{cases} \tag{15}$$

The constant term P is substituted with the real variable function $P(s)$ to maintain the equilibrium between the ant's random search and the path details in the induction function during the random search process, while phenomena are rising. Where P_1, $P2, P_3$ are the function values corresponding to different ranges of s, and S_1, $S2, S_3$ are the threshold points.

Dynamic Evaporation Component Approach: The pheromone's evaporation factor is a constant in the fundamental HACO algorithm. Large-scale problems have almost zero undiscovered path phenomena, and the value is directly related to the speed of convergence and global search capabilities of the ACO algorithm. In the process of enhancing the ACO algorithm's capacity for broad search, the dynamic evaporation rate technique significantly improves the convergence. Three distinct decay models are available to better investigate the evaporation rate decay model,such as the curve, line, and scale decay models. A series of experiments is used to determine the curve decay model. The following Equation (16) describes the definition of the curve decay model.

$$\rho(s) = \frac{S\times(\tau_{Maxi}-\tau_{Mini})\times s}{S-1} + \frac{S\times\tau_{Mini}-\tau_{Maxi}}{S-1} \quad (16)$$

Where τ_{Maxi} and τ_{Mini} are the pheromone's upper and lower limits, the terms "s" and "S" stand for the present iteration and the maximum number of iterative times.

Symmetrical Boundary Mutation Approach: The distribution of the collected data will probably tend to the normal distribution as the number of samples approaches infinity, according to mathematical statistics. The center area has the boundary path that shows the advantages and disadvantages of routes. Boundary balance indicates that boundary mutation only occurs inside this border; crossover and boundary mutation do not occur. According to the experimental findings, the enhanced mutation approach greatly increases both the effectiveness and efficiency of mutations.

Fig.4. represents the Ant Colony Optimization (ACO) algorithm workflow. It begins with parameter initialization and updating the taboo table with OD points. Ants traverse possible paths, depositing pheromones to mark favorable routes. The process iteratively updates the current optimal path through repeated rounds, reinforcing better solutions. After multiple iterations, the best path is identified and output as the final result. This bio-inspired optimization

method mimics ant foraging behavior and is widely applied in routing, scheduling, and path optimization problems.

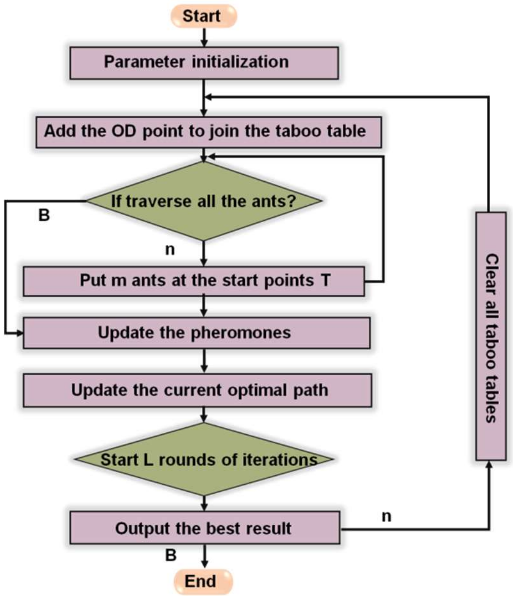

Fig.4. Flowchart of the HACO Algorithm

The accuracy of the selected feature subset for each ant is assessed using a Logistic Regression classifier. Every characteristic is given a pheromone level that denotes its significance. Based on pheromone intensity, several artificial agents (ants) actively select attributes. When an observation receives more pheromone deposits, the significance of qualities that lead to increased accuracy is reinforced. Until the optimal selection of features is found, the algorithm iterates several times, progressively improving the selection procedure. Algorithm 1 indicates the CA-HACO-LF algorithm.

Algorithm 1: CA-HACO-LF

Initialize the preprocessing metrics (data, tokens, emmas)

$$data = load\_kaggle\_drug\_data()$$
$$data = normalize\_text(data)$$
$$tokens = tokenize(data)$$
$$tokens = remove\_stopwords(tokens)$$
$$lemmas = lemmatize(tokens)$$

Initialize the feature extraction metrics (features, similarity_scores)

$$features = extract\_ngrams(tokens)$$
$$similarity\_scores = compute\_cosine\_similarity(features)$$

$Def\ CA - HACO - LF$

$$contextual\_features = add\_contextual\_data(data)$$

$$rf\_model = train\_random\_forest(optimal\_features)$$
$$lr\_model = train\_logistic\_regression(optimal\_features)$$
$$final\_predictions = majority\_vote(rf\_model.predict(test\_data), lr\_model.predict(test\_data))$$
For iteration in range$(max\_iterations)$:
For ants in ants:
$$selected\_features = select\_features\_with\_pheromone(ant, features)$$
$$Accuracy = evaluate\_features\_with\_logistic\_regression(selected\_features)$$
$$update\_pheromones(selected\_features, Accuracy)$$
$$optimal\_features = get\_best\_feature\_subset()$$
Output: $evaluate\_model(final\_predictions, true\_labels)$

The CA-HACO-LF model combines contextual information with enhanced ML to forecast drug-target interactions. By employing advanced optimization techniques and real-world pharmacological datasets to improve prediction accuracy, CA improves flexibility in response to changing medical data, the LF classifier increases classification accuracy, and HACO selects the most pertinent characteristics. Cosine similarity assesses semantic linkages in drug descriptions, and the CA-HACO-LF model facilitates intelligent drug recommendation.

14 The integrated LR and RF method is compared with numerous optimizations, including Genetic Algorithm (GA), Particle Swarm Optimization (PSO), Simulated Quantum Annealing Optimization (SQAO), and Ant Colony Optimization (ACO), to identify the effective performance.

GA: GA is inspired by evolution and natural selection, with binary chromosomes representing traits. Generations change through mutation, crossover, and selection, with fitness ratings influencing final features, evaluated by LR.

PSO: PSO represents potential features in a feature region using particles, deriving inspiration from coral reefs and bird flocks. Top features are chosen for the hybrid classifier by iteratively evaluating each particle's population using LR and selecting the global best component.

SQAO: To facilitate effective exploration and convergence towards optimal classifying features, SQAO, a quantum mechanics-based technique, iteratively converts features using simulated heating, and frequently accepts lower quality states to avoid local optima.

ACO: An optimization based on the ACO approach was developed, inspired by ants' foraging
35 behaviors. Its application in feature selection assists in selecting the most instructive features for the classification task.

After each method's feature selection is finished, the chosen features are utilized to train two classifiers, such as RF and LR. A hybrid decision is produced by averaging the forecasts in an effort to better generalize by capturing both non-linear and linear patterns.

Results and Discussions

The primary concern in the research involves the advancement of drug-target interaction forecasting in drug discovery. The implementation is performed by Python with 32GB RAM. Performance evaluations of the model, the comparative evaluations, and the optimization comparisons are demonstrated in this section.

Evaluations Performance

This section provides effective performance with Cosine Similarity Heatmap, Word Cloud Visualizations for medicine uses, side effects, and composition, Frequent Words Used in Medicine, Frequent Medicine Names, and Confusion Matrix.

- ✓ **Cosine Similarity Heatmap:** A heatmap that compares materials, values, or other vector representations by using color to show the cosine relationship between pairings of vectors is called a cosine similarity heatmap.

Fig.5. is a heatmap showing similarity scores between different medicines. Dark red indicates higher similarity (close to 1), while blue shows lower similarity (near 0). It helps visualize relationships, overlaps, or substitutability among various drugs efficiently.

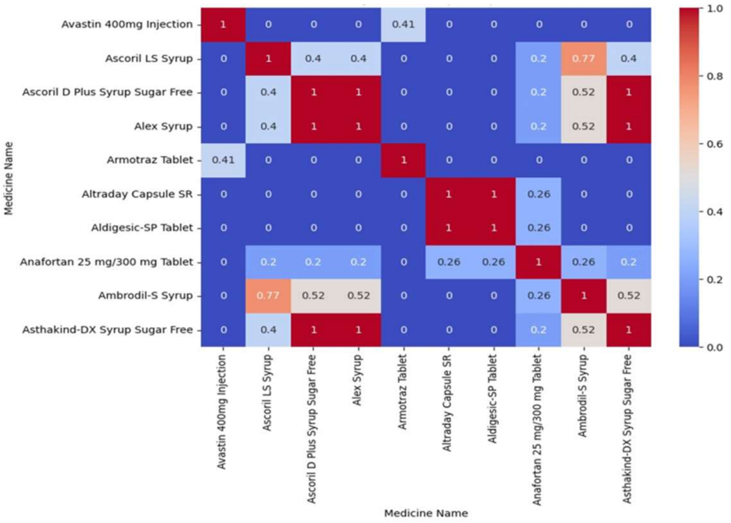

Fig.5. Visual Representation of Cosine Similarity Heatmap

All medications are identical to one another; hence, the diagonal values are always 1. The similarity between several medications is indicated by off-diagonal measurements. Areas that are

warm or red show a high degree of similarity. Blue regions show a slight similarity. For instance, "Ascoril LS Syrup" shares strong similarities with "Ascoril D plus Syrup Sugar-Free" and "Alex Syrup," indicating that the applications are identical.

- ✓ **Word Cloud Visualizations:** The visual representations of text data, known as word cloud visualizations, show the frequency or significance of each word in a text by displaying its size. The cloud visualizations for medicine uses, side effects, and compositions are below.

Medicine Uses: Using a collection of information about medicine, it develops a Word Cloud visualization to show the most common words in the "Uses" category (Figure 6).

Figure 6 (a) is a word cloud representing common medical terms related to drug usage and conditions. Larger words like pain, treatment, relief, and neuropathic indicate higher frequency, highlighting key therapeutic purposes such as pain management, chest issues, hypertension, and heart-related treatments.

Side Effects of Medicines: The word cloud visualization is produced by the investigation to show the most common terms in the "Side effects" category of a dataset that contains information on medications.

Fig.6. is a word cloud of drug side effects, where larger words like nausea, pain, dizziness, vomiting, and headache represent the most frequent adverse effects. Smaller terms indicate less common issues, highlighting the broad spectrum of potential medication reactions.

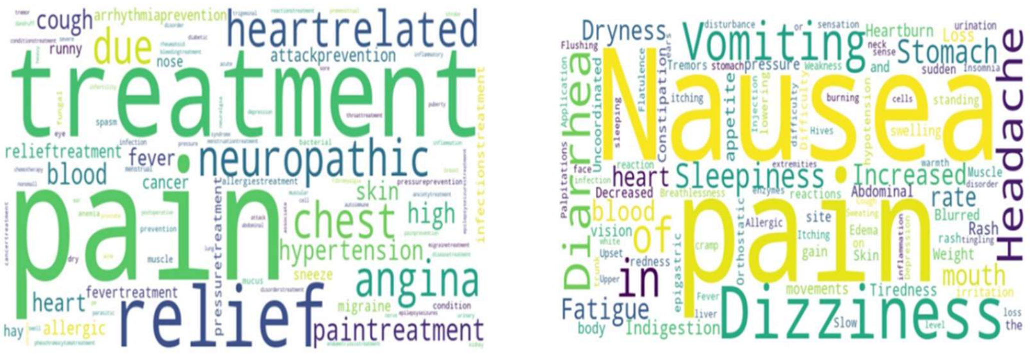

(a) (b)

Fig.6. Word Cloud Visualizations for **(a)**Medicines uses**(b)**Side Effects of Medicines

Composition of Medicines: To highlight the most common words in the "Composition" category of a dataset with information about medicine, the research establishes a Word Cloud visualization.

Fig.7. is a word cloud of drug compositions and dosages. Prominent terms like Paracetamol, Diclofenac, Aceclofenac, 100mg, and 325mg indicate frequently used medicines and strengths, while smaller words represent less common compounds, highlighting the diversity of pharmaceutical formulations.

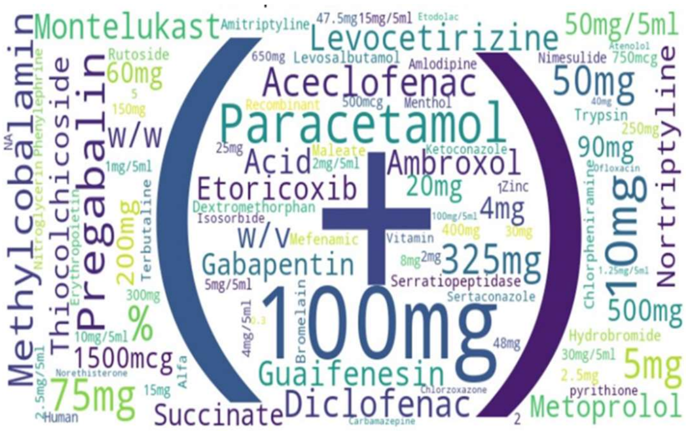

Fig.7. Word Cloud Visualizations for Composition of Medicines

- ✓ **Frequent Words Used in Medicine:** Processing an image, extracting pertinent information, and producing a visual representation of the retrieved data are the primary objectives. Techniques, including image segmentation, extracting features, and statistical evaluation, have been used in the process.

Figure 8 (a) is a bar chart showing medicine usage word frequency. Pain and treatment dominate with the highest counts, followed by relief, neuropathic, angina, and heart-related. This highlights major therapeutic focuses, emphasizing pain management and treatment as primary healthcare concerns.

According to the outcomes, "pain" and "treatment" are the most commonly used words in medication usage, demonstrating the significance of drug descriptions. A focus on addressing chronic and cardiovascular diseases is suggested by terms like "relief," "neuropathic," and "chest," which represent common therapeutic areas that drugs address.

- ✓ **Frequent Medicine Names:** The investigation examines a dataset containing medication names.

Fig.8. illustrates the frequency of prescribed medicines across different categories, including creams, syrups, tablets, capsules, and vaccines. Among them, Episert Cream, Monoguard Cream, Benadryl Syrup, and Sertaspore Cream were prescribed most frequently, with a count of three each. In comparison, the remaining medicines, such as Mibeta SR 40 Tablet and Pregalin Forte Capsule, showed relatively lower frequencies of two.

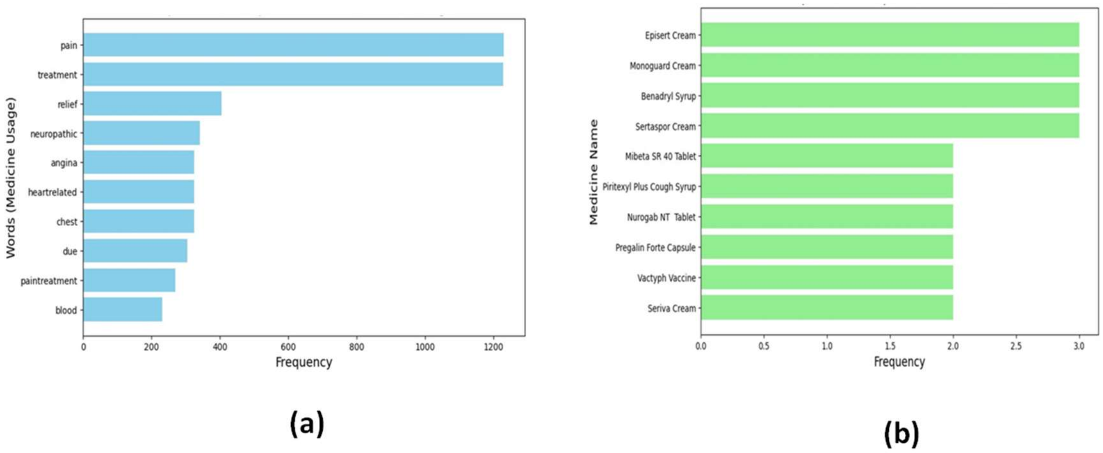

Fig.8.(a) Frequent Words Used in Medicine **(b)**Frequent Medicine Names

Episert Cream, Monoguard Cream, and Benadryl Syrup are among the top ten most often referenced medication names. The existence of tablets, creams, syrups, and vaccinations suggests a wide variety of formulations utilized, mostly for pain management, topical treatment, respiratory relief, and preventative care across many therapeutic categories.

- ✓ **Confusion Matrix:** By classifying the actual and expected classes, a confusion matrix demonstrates the proportion of accurate (TP - True Positives and TN - True Negatives) and inaccurate forecasts (FP – False Positives and FN – False Negatives). The confusion matrix for the CA-HACO-LF method was obtained from the data of drug discovery.

7

Fig.9. illustrates the classification performance across five classes. Most predictions align with true labels, showing high accuracy. Class 1 achieved the best results with 324 correct predictions. Minor misclassifications occurred in Classes 0 and 2, where some samples were incorrectly classified. Overall, the model demonstrates strong predictive performance with minimal errors across all categories.

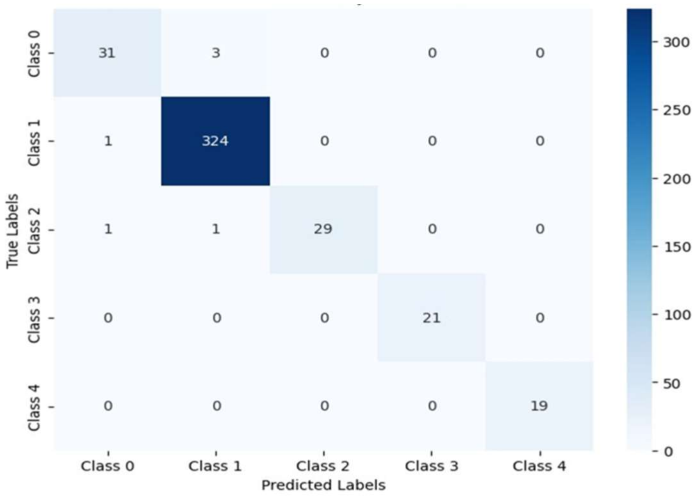

Fig.9. Confusion Matrix for Drug Discovery

The confusion matrix shows the CA-HACO-LF model classified five distinct medical diseases, including Class 0: Cough, Class 1: Pain, Class 2: Cancer, Class 3: Fever, and Class 4: Infections. Using only a small amount of misclassification, the model accurately identified 324 instances of "Pain" (Class 1). Precisely one case was incorrectly identified as "Cough" (Class 0), and another as "Cancer" (Class 2).

Comparative Evaluations

Comparison between the proposed CA-HACO-LF and existing techniques, such as Bidirectional Encoder Representations from Transformers BERT [27], Extreme Gradient Boosting (XGBoost) [28], Random Forest (RF) [28] and K-Nearest Neighbour (KNN) [28], Multi Task Deep Neural Network (DNN) [29]is demonstrated in the research by employing numerous performance metrics, like accuracy, precision, recall, F1 score, F2 score, Root Mean Squared Error (RMSE), Cohen's Kappa, AUC ROC curve, Mean Absolute Error (MAE), and Mean Squared Error (MSE). Table 2 examines the metric's definitions and equations. Table 3 shows the comparison of various existing and CA-HACO-LF methods' performances in drug discovery.

Table 2. Definitions and Equations of the Performance Matrices

Metrics	Definitions	Equations
Accuracy	The proportion of accurate true positive and true negative forecasts overall.	$Accuracy = \frac{TP + TN}{TP + TN + FP + FN}$
Precision	The proportion of real positive predictions among all the positive predictions provided by the model.	$Precision = \frac{TP}{TP + FP}$
Recall		

8
41

F1 Score	It is the harmonic mean of precision and recall. It measures the balance between both metrics.	$F1\ score = 2 \times \frac{Precision \times Recall}{Precision + Recall}$
Cohen's Kappa	It evaluates the probability of random agreement while calculating the level of consistency between the expected and actual labels.	$\kappa = \frac{Pr(x) - Pr(f)}{1 - P(f)}$
AUC ROC	It determines whether the model allows for differentiation among various classes using probability outputs. Having the highest AUC shows efficient results in forecasts.	$\text{AUC ROC} = \int_0^1 \text{TP(FP)dFP} = \int_0^1 \text{TP}(\text{FP(a)})\text{da}$
F2 Score	A variant of the F-score that prefers recall above precision, which makes it effective for instances when inaccurate results are more important than incorrect positives.	$F2\ SCore = 5 \times \frac{Precision \times Recall}{(4 \times Precision) + Recall}$
RMSE	It measures the average magnitude of the errors that receive large errors with weight due to.	$RMSE = \sqrt{\frac{1}{N}\sum_{j=1}^{N}(b_j - \hat{b}_j)^2}$
MSE	It evaluates the average magnitude of forecast errors without taking motion into consideration.	$MSE = \frac{1}{N}\sum_{j=1}^{N}\lvert b_j - \hat{b}_j \rvert$
MAE	Compared to MSE, it is more robust to severe errors as it measures the absolute variation among anticipated and actual values.	$MAE = \frac{1}{N}\sum_{j=1}^{N}\lvert B_j - \hat{B}_j \rvert$

Table 3. Comparison of Various Exiting and CA-HACO-LF Methods

**Parameter**	**BERT[27]**	**XGBoost [28]**	**KNN [28]**	**RF [28]**	**MultiTask DNN [29]**	**CA-HACO-LF [Proposed]**
Accuracy	0.97	0.80	0.77	0.72	0.88	0.986
Precision	0.968	0.73	0.83	0.63	0.84	0.985
Recall	0.963	0.87	0.62	0.87	0.86	0.986
F1Score	0.965	0.79	0.71	0.73	0.85	0.985
Cohen's Kappa	0.9363	0.61	0.53	0.45	-	0.9658

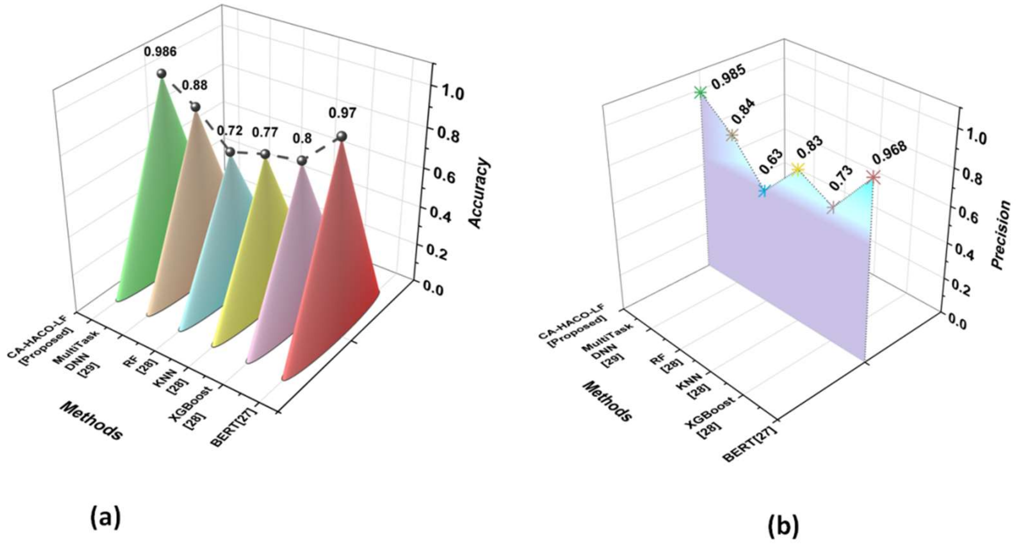

Fig.10. Results of (a) Accuracy and (b) Precision

Fig.10. (a-b) depict the accuracy and precision results. Whereas the accuracy determined by the proposed CA-HACO-LF method is 0.986, existing methods like BERT, XGBoost, KNN, RF and MultiTask DNN provided accuracy of 0.97, 0.80, 0.77, 0.72 and 0.88 Based on the results, the proposed method showed an improved result compared to than the existing techniques in drug discovery accuracy. Precision in CA-HACO-LF is 0.985, BERT is 0.968, XGBoost is 0.73, KNN is 0.83, RF is 0.63 and Multi Task DNN is 0.84. Findings of the precision indicated that the performance of the proposed CA-HACO-LF surpasses all other existing techniques in drug discovery.

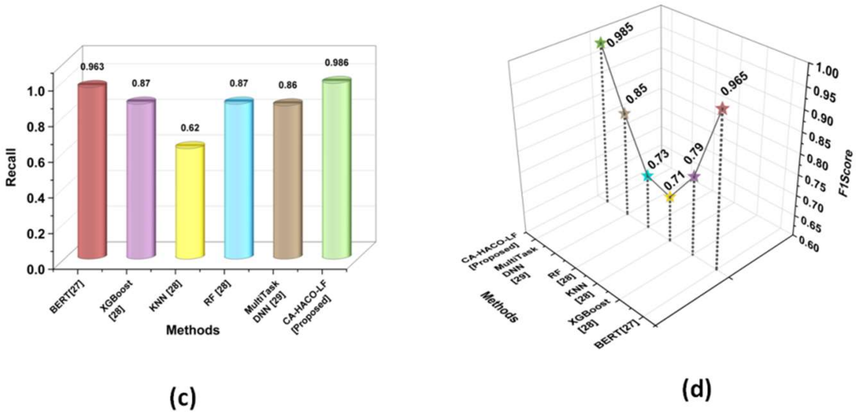

Figure 11: Evaluation Outcomes of (a) Recall and (b) F1 score

Fig.11. (a-b) represented the recall and F1 score outcomes. Recall rate of BERT (0.963), XGBoost (0.87), KNN (0.62), RF (0.87), Multi Task DNN (0.86) and CA-HACO-LF (0.986) techniques are explored. According to the results, the proposed method provides significant outcomes with an enhanced recall rate for drug discovery performance. The F1 score of RF is 0.73, BERT is 0.965, XGBoost is 0.79, KNN is 0.71, Multi Task DNN is (0.85) and the proposed
36 F1 score is 0.985. Thus, the research results indicated that the proposed method is superior to all other existing drug discovery forecasting and classification approaches.

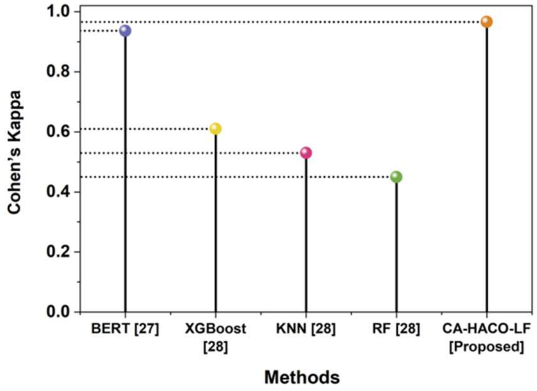

Fig.12. Estimation of Cohen's Kappa with Proposed and Existing Techniques

The Cohen's Kappa metric outcomes are displayed in Fig.12. The CA-HACO-LF technique provides a 0.9658 Cohen's Kappa rate, while XGGBoost has 0.61, RF has 0.45, BERT has
26 0.9363 and KNN has 0.53. Thus, the results of the research showed that the improved result of the proposed model ismore significant in the discovery of drugs than existing techniques.Table 4 determined the evaluation outcomes of the BERT [27] model and the CA-HACO-LF model in drug discovery processes.

Table 4. Evaluation outcomes of the BERT [27] model and the CA-HACO-LF model in drug discovery processes

Parameter	BERT[27]	CA-HACO-LF [Proposed]
AUC ROC Score	0.9682	0.9943
F2 Score	0.9682	0.9859
MSE	0.0318	0.0209
RMSE	0.1785	0.1446
MAE	0.0318	0.0162

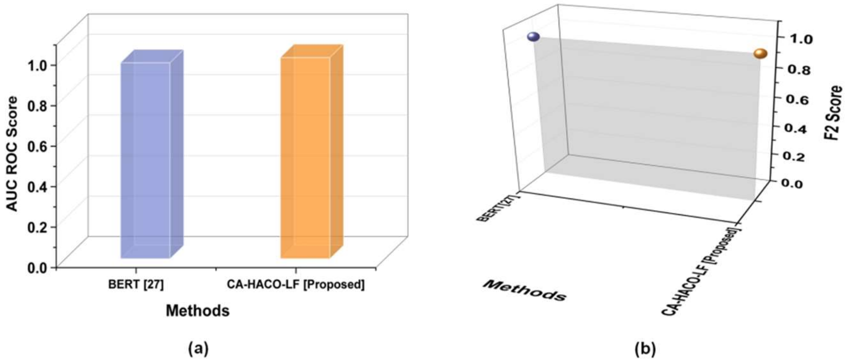

Fig.13. Findings of the CA-HACO-LF and BERT techniques with (a) AUC ROC Score and (b) F2 Score

Findings of the proposed and existing methods' AUC ROC Score and F2 score are explored in Fig.13.(a-b). BERT with 0.9682 of AUC ROC and 0.9682 of F2 Score, CA-HACO-LF with 0.9943 of AUC ROC and 0.9859 of F2 Score are provided. Whereas the proposed model exceeds the existing technique in the drug discovery process with significant efficiency in both AUC ROC and F2 Score.

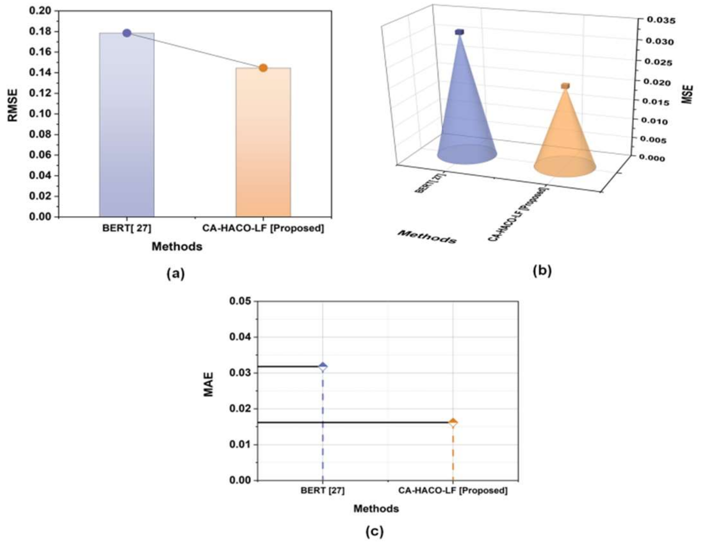

Fig.14. Comparison Outcomes of Error Metrics (a) RMSE, (b) MSE and (c) MAE

Fig.14.(a-c) demonstrated the results of the proposed CA-HACO-LF and the existing BERT method's RMSE (CA-HACO-LF-0.1446, and BERT-0.1785), MSE (CA-HACO-LF-0.0209, and BERT-0.0318) and MAE (CA-HACO-LF-0.0162, and BERT-0.0318) outcomes. The CA-HACO-LF is enhanced more significantly than the BERT approach in terms of fewer error outcomes.

Optimization Comparison

The research provides the comparison of LR and RF with various optimizations such as GA, PSO, SQAO, and ACO. Table 5 represents the optimization results of LR and RF in the drug discovery process.

Table 5. Comparison of LR and RF with Various Optimizations

Parameters	GA with LR and RF	PSO with LR and RF	SQAO with LR and RF	ACO with LR and RF
Accuracy (%)	91	95	96	99
Precision (%)	92	96	96	99
Recall (%)	91	95	96	99
F1Score (%)	90	95	96	99

According to the results, the LR and RF with ACO performance was more significant than other optimization integrated with LR and RF techniques in terms of accuracy (99%), precision (99%), recall (99%), and F1 Score (99%).

Whereas BERT [27] appeared to be effective at interpreting contextual language, it required a lot of processing power and had difficulty integrating complex biological features. The XGBoost [28] and RF [28] were over-fit with medical information and lacked the awareness of context. With high-dimensional data, which is typical in drug discovery, KNN [28] performed poorly and was affected by feature scaling. Additionally, feature redundancy was not sufficiently addressed by these models, which impacted the forecasting accuracy. By incorporating HACO for optimal selection of features and minimizing noise and duplication, the proposed CA-HACO-LF resolves the existing limitations. To improve robustness and generality, it integrates the LF method. Furthermore, by greatly enhancing prediction accuracy and flexibility in real-world pharmaceutical datasets, the incorporation of CA learning and semantic similarity algorithms facilitates better comprehension of medication descriptions.Finding the most effective solutions is demanded with the GA method, particularly when dealing with technical drug discovery issues with large search fields. PSO underestimated the global optimum when it converged to local optima too rapidly. SQAO was still not widely utilized in drug discovery, and its implementation was more complicated than other optimizations. The ensemble classifier used the highly relevant features that ACO had effectively chosen to generate reliable and broadly applicable outputs. In all class distributions, the result demonstrates superior adaptability and intellectual capacity.

In the fields of healthcare, biotechnology, and customized treatment planning, its precise prediction of drug-target interactions facilitates quicker drug development, lowers expenses and failures, and can be modified for medical text mining, toxicity prediction, and pharmaceutical genomics.

Ablation Research Results

The ablation research evaluates the effectiveness of each component in the proposed CA-HACO-LF model. The full model achieved the highest performance (Accuracy: 0.986, F1 Score: 0.986), demonstrating the benefit of combining context-aware learning, ACO-based feature selection, and logistic forest classification. Removing the context-aware module or ACO feature selection led to noticeable drops in accuracy and F1 score, highlighting their importance in enhancing prediction quality. Eliminating cosine similarity further reduced semantic understanding of drug descriptions. Table 6 using only RF or LR showed significantly lower performance, confirming that the hybrid integration is critical for superior drug-target interaction prediction.

Table 6. Ablation Results of The Proposed CA-HACO-LF Model

Model Variant	Accuracy	Precision	Recall	F1 Score
**Proposed CA-HACO-LF (Full Model)**	**0.986**	**0.985**	**0.988**	**0.986**
− Without Context-Aware	0.979	0.977	0.981	0.979
− Without ACO Feature Selection	0.973	0.971	0.974	0.973
− Without Cosine Similarity	0.971	0.970	0.971	0.971
Random Forest only	0.964	0.963	0.964	0.964
Logistic Regression only	0.952	0.951	0.952	0.952

Conclusions

The research focused on improving predictive accuracy for drug-target interactions. A dataset of over 11,000 drug entries was collected and pre-processed using text normalization (lowercasing, punctuation removal, elimination of numbers and spaces), stop word removal, tokenization, and lemmatization to ensure data quality. Significant features were extracted using N-grams and Cosine Similarity. The CA-HACO-LF model, which combines RF and LR with HACO, was used for classification. The evaluation results implemented in Python show that CA-HACO-LF

3 achieved an accuracy of 98.6%, precision of 0.985, recall of 0.986, F1 score of 0.985, F2 score of 0.9859, RMSE of 0.1446, MSE of 0.0209, MAE of 0.0162, Cohen's Kappa of 0.9658, and AUC-ROC of 0.9943. These outcomes indicate improved performance relative to standard RF and LR models; however, these results are specific to the dataset and experimental conditions used in this study. It is important to acknowledge the limitations of this work: the model's generalizability to other drug-target datasets has not been fully validated, and performance may vary with different data distributions. Future research should include larger and more diverse datasets to further assess the robustness and applicability of the CA-HACO-LF approach.

Limitations and Future Scopes

The CA-HACO-LF model requires a significant amount of computing power and it is limited in its ability to manage huge, diverse datasets. Future research should therefore focus on optimizing the computational efficiency of the model, possibly through lightweight architectures, parallelized frameworks, or integration with cloud-based and edge computing environments.Further investigations proceed in requiring directions with wider clinical deployment and application in unique illness medication discovery. While the CA-HACO-LF model demonstrates strong predictive performance, several practical challenges remain for real-world deployment. First, the computational cost associated with hybrid optimization and ensemble learning can limit scalability in resource-constrained environments, requiring efficient model compression or cloud-based deployment strategies. Second, integration into pharmaceutical pipelines demands compatibility with diverse biomedical data formats and regulatory compliance, which can pose technical and administrative hurdles. Finally, real-world adoption requires interpretability and ease of deployment so that domain experts can trust and utilize the system effectively in clinical and industrial contexts. Addressing these aspects will be crucial for translating the promising results of this research into practical applications.

Funding

This research work was supported by the National Research Foundation of Korea(NRF) grant funded by the Korea government (MSIT) (NRF-2023R1A2C1005950).

2

Data Availability

All datasets used in this study, including those sourced from Kaggle (26.https://www.kaggle.com/datasets/singhnavjot2062001/11000-medicine-details), are publicly available and come with licenses that grant permission for research use.

Reference

1. Adelusi, T.I. et al. Molecular modeling in drug discovery. *Informat. Med. Unlocked* 29, 100880 (2022). https://doi.org/10.1016/j.imu.2022.100880
2. Shaker, B. et al. In silico methods and tools for drug discovery. *Comput. Biol. Med*. 137, 104851 (2021). https://doi.org/10.1016/j.compbiomed.2021.104851
3. Walters, W.P. and Barzilay, R.,. Critical assessment of AI in drug discovery. *Expert opinion on drug discovery*, *16*(9), pp.937-947 (2021). https://doi.org/10.1080/17460441.2021.1915982
4. Vijayan, R.S.K., Kihlberg, J., Cross, J.B. & Poongavanam, V. Enhancing preclinical drug discovery with artificial intelligence. *Drug Discov. Today* 27, 967–984 (2022). https://doi.org/10.1016/j.drudis.2021.11.023
5. Power, H., Valtchev, P., Dehghani, F. et al. A. Strategies for senolytic drug discovery. *Aging Cell 22,* e13948 (2023). https://doi.org/10.1111/acel.13948
6. Ma, Z., Bolinger, A.A. & Zhou, J. RIPTACs: a groundbreaking approach to drug discovery. *Drug Discov*. Today 28, 103774 (2023). https://doi.org/10.1016/j.drudis.2023.103774
7. Gangwal, A. & Lavecchia, A. Unlocking the potential of generative AI in drug discovery. *Drug Discov. Today* 103992 (2024). https://doi.org/10.1016/j.drudis.2024.103992
8. Obaido, G., Mienye, I.D., Egbelowo, O.F., Emmanuel, I.D., Ogunleye, A., Ogbuokiri, B., Mienye, P. & Aruleba, K. Supervised machine learning in drug discovery and development: Algorithms, applications, challenges, and prospects. *Mach. Learn. Appl.* 17, 100576 (2024). https://doi.org/10.1016/j.mlwa.2024.100576
9. Abbas, M.K.G., Rassam, A., Karamshahi, F., Abunora, R. & Abouseada, M. The role of AI in drug discovery. *ChemBioChem* 25, e202300816 (2024). https://doi.org/10.1002/cbic.202300816

10. Rasul, H.O., Ghafour, D.D., Aziz, B.K., et al. Decoding drug discovery: Exploring A-to-Z in silico methods for beginners. *Appl. Biochem. Biotechnol.* 197, 1453–1503 (2025). https://doi.org/10.1007/s12010-024-05110-2
11. Yoo, J., Jang, W. & Shin, W.H. From part to whole: AI-driven progress in fragment-based drug discovery. *Curr. Opin. Struct. Biol.* 91, 102995 (2025). https://doi.org/10.1016/j.sbi.2025.102995
12. van Tilborg, D., Brinkmann, H., Criscuolo, et al. Deep learning for low-data drug discovery: Hurdles and opportunities. *Curr. Opin. Struct. Biol.* **86**, 102818 (2024). https://doi.org/10.1016/j.sbi.2024.102818
13. Lin, M., Cai, J., Wei, Y., et al. MalariaFlow: A comprehensive deep learning platform for multistage phenotypic antimalarial drug discovery. *Eur. J. Med. Chem.* **277**, 116776 (2024). https://doi.org/10.1016/j.ejmech.2024.116776
14. Wang, Y., Chen, X., Tang, N., et al. Boosting clear cell renal carcinoma-specific drug discovery using a deep learning algorithm and single-cell analysis. *Int. J. Mol. Sci.* **25**, 4134 (2024). https://doi.org/10.3390/ijms25074134
15. Wu, J., Xiao, Y., Lin, M., et al. DeepCancerMap: A versatile deep learning platform for target- and cell-based anticancer drug discovery. *Eur. J. Med. Chem.* **255**, 115401 (2023). https://doi.org/10.1016/j.ejmech.2023.115401
16. Qian, Y., Ni, W., Xianyu, X., et al. Deep learning for drug discovery: A case study on non-small cell lung cancer with EGFR T790M mutation. *Pharmaceutics* **15**, 675 (2023). https://doi.org/10.3390/pharmaceutics15020675
17. Isert, C., Atz, K. & Schneider, G. Structure-based drug design with geometric deep learning. *Curr. Opin. Struct. Biol.* **79**, 102548 (2023). https://doi.org/10.1016/j.sbi.2023.102548
18. Wang, C.T. & Chen, B.S. Drug discovery for periodontitis treatment based on big data mining, systems biology, and deep learning methods. *SynBio* **1**, 116–143 (2023). https://doi.org/10.3390/synbio1010009
19. Sun, J., Ni, Q., Jiang, F., et al. J. Discovery and validation of traditional Chinese and Western medicine combination antirheumatoid arthritis drugs based on machine learning (random forest model). *Biomed. Res. Int.* **2023**, 6086388 (2023). https://doi.org/10.1155/2023/6086388

20. Zhou, Y. Antistroke network pharmacological prediction of XiaoshuanTongluo recipe based on drug-target interaction using deep learning. *Comput. Math. Methods Med.* **2022**, 6095964 (2022). https://doi.org/10.1155/2022/6095964

21. Murali, V., Muralidhar, Y.P., Königs, C., et al.. Predicting clinical trial outcomes using drug bioactivities through graph database integration and machine learning. *Chem. Biol. Drug Des.* 100, 169–184 (2022). https://doi.org/10.1111/cbdd.14092

22. Shen, J. & Valagolam, D. A systematic implementation of machine learning algorithms for multifaceted antimicrobial screening of lead compounds. *Med. Sci. Forum* **12**, 6 (2022). https://doi.org/10.3390/eca2022-12751

23. Abbas, K., Abbasi, A., Dong, S., et al. Application of network link prediction in drug discovery. *BMC Bioinformatics* **22**, 1–21 (2021). https://doi.org/10.1186/s12859-021-04082-y

24. El-Behery, H., Attia, A.F., El-Fishawy, et al. Efficient machine learning model for predicting drug-target interactions with case study for COVID-19. *Comput. Biol. Chem.* **93**, 107536 (2021). https://doi.org/10.1016/j.compbiolchem.2021.107536

25. Margulis, E., Dagan-Wiener, A., Ives, R.S., Jaffari, S., Siems, K. & Niv, M.Y. Intense bitterness of molecules: Machine learning for expediting drug discovery. *Comput. Struct. Biotechnol. J.* 19, 568–576 (2021). https://doi.org/10.1016/j.csbj.2020.12.030

26. Singh, N. 11,000 medicine details. *Kaggle* (2023). https://www.kaggle.com/datasets/singhnavjot2062001/11000-medicine-details

27. Nagalakshmi, R., Khan, S.B., Mahesh, T.R., et al. Enhancing drug discovery and patient care through advanced analytics with the power of NLP and machine learning in pharmaceutical data interpretation. *SLAS Technol.* 31, 100238 (2025). https://doi.org/10.1016/j.slast.2024.100238

28. Saha, S., Chatterjee, P., Halder, A.K., et al. ML-DTD: Machine learning-based drug target discovery for the potential treatment of COVID-19. *Vaccines* 10, 1643 (2022). https://doi.org/10.3390/vaccines10101643

29. Islam, S., Lincoln, S.S. & Rupa, M.A. AI-driven pharmacology: Leveraging machine learning for precision medicine and drug discovery. *Int. J. Comput. Appl.* 975, 8887 (2023).
